# Supplementary material for: A-FABP in Metabolic Diseases and the Therapeutic Implications: An Update
Source: Int J Mol Sci. 2021 Aug 30;22(17):9386. doi: 10.3390/ijms22179386 (PMC8456319; doi:10.3390/ijms22179386)
Supplement: Supplementary file 1 [file ijms-22-09386-s001.zip › ijms-1364365-supplementary.pdf]

Central Illustration. Diseases associated with A-FABP and benefits of A-FABP inhibition

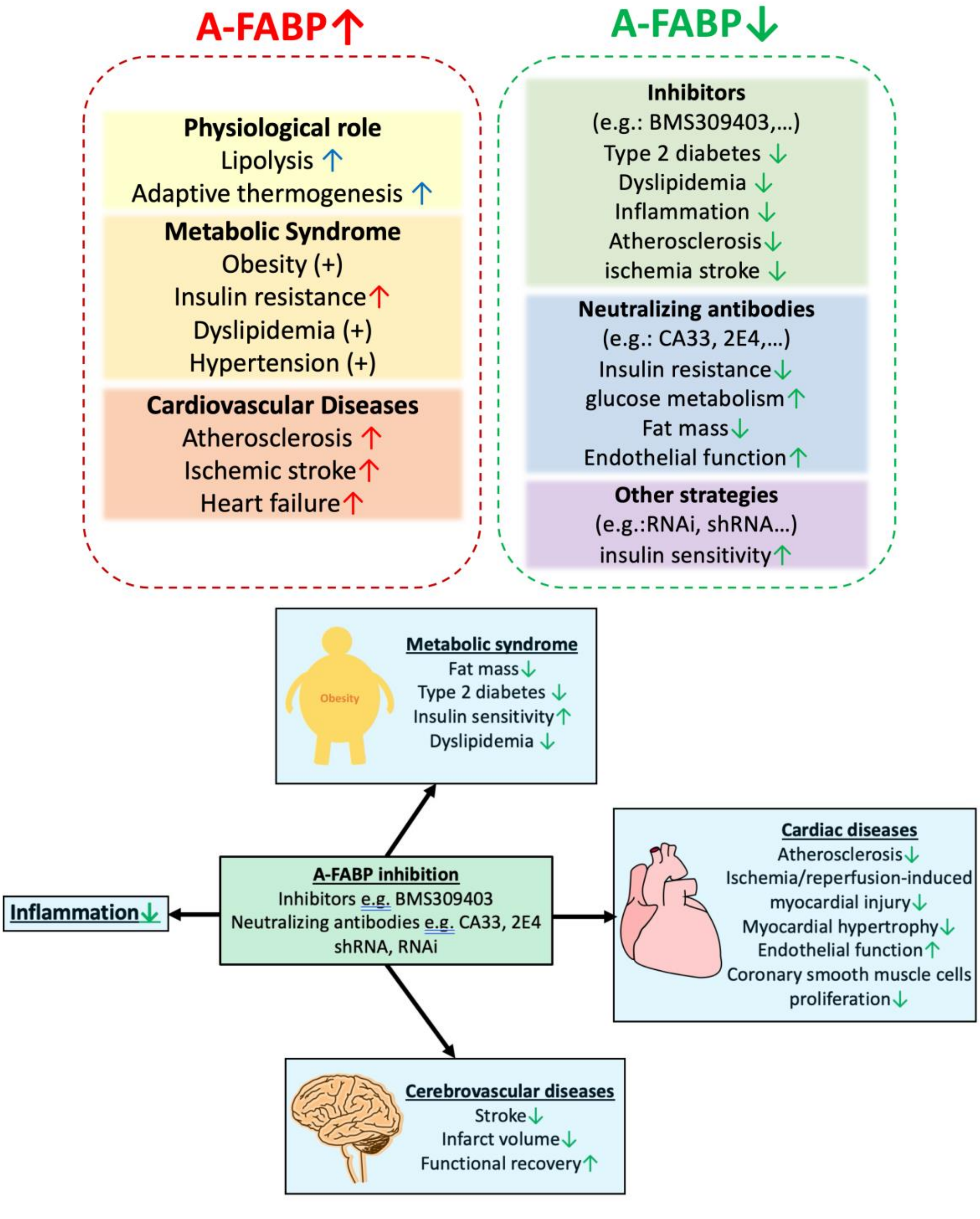

↑: stimulation; ↓ suppression; (+) positive correlation
